# Supplementary material for: Transcriptomic Studies Suggest a Coincident Role for Apoptosis and Pyroptosis but Not for Autophagic Neuronal Death in TBEV-Infected Human Neuronal/Glial Cells
Source: Viruses. 2021 Nov 10;13(11):2255. doi: 10.3390/v13112255 (PMC8620470; doi:10.3390/v13112255)
Supplement: Supplementary file 1 [file viruses-13-02255-s001.zip › viruses-1416846-supplementary.pdf]

viruses MDPI

Supplementary Materials: The following are available online at www.mdpi.com/xxx/s1, Table S1: Primers pairs used for qRT-PCR analyses. Table S2: TBEV-induced differential expression of apoptotic genes in human neuronal/glial cells. Table S3: TBEV-induced differential expression of autophagic genes in human neuronal/glial cells.

Table S1. Primers pairs used for qRT-PCR analyses.

Gene name	3' primer	5' primer	Origin
AIM2	GCTGCAC-CAAAAGTCTCTCCTC	CTGCTTGCCTTCTT-GGGTCTCA	Retrieve from https://www.ori-gene.com
ATG3	ACATGGCAATGGGC-TACAGG	CTGTTTGCAC-CGCTTATAGCA	Yu et al. 2018
BECN1	ACCTCAGCCGAA-GACTGAAG	AACAGCGTTTGTAG-TTCTGACA	Sharma et al. 2011
CASP1	TTTCCG-CAAGGTTCGATTTTCA	GGCATCTGCGCTCTAC-CATC	Liu et al. 2018
GSDMD	GGTTCTG-GAAACCCCGTTAT	CCAGGTGTTAGGGTCCAC AC	Designed * from Acc. No NM_024736.7
HPRT1	GGACTAATTATGGACA GGACT	GCTCTTCAGTCTGA-TAAAATCTAC	Fares et al. 2020
TNFSF10	AGCAATGCCACTTTT-GGAGT	TTCACAGTGCTCCTG-CAGTC	Lu et al. 2018
TNF	AGATGATCTGACTGCCT GGG	TGCTT-GTTCCTCAGCCTCTT	Melchjorsen et al. 2010
TNFRSF1A	GAGAGGCCA-TAGCTGTCTGG	GTTCCTTTGTGGCACTT-GGT	Designed * from Acc. No NM_001065.4
TNFRSF10A	AGAGAGAAGTCCCTG-CACCA	GTCACTCCAGGGCG-TACAAT	Designed * from Acc. No NM_003844.4
TNFRSF10B	TGCAGCCGTAGTCTT-GATTG	TCCTG-GACTTCCATTTCCTG	Designed * from Acc. No NM_003842.5

*Designed using primer designer from the website: https://www.bioinformatics.nl/cgi-bin/primer3plus/primer3plus.cgi.

Table S2. TBEV-induced differential expression of apoptotic genes in human neuronal/glial cells. (PCR array data, 72hpi). Up- and down-regulated genes (threshold of 3) are indicated in bold and underscored.

Gene symbol	Ct		Fold Change
	NI	TBEV	
ABL1	35	35	1.18
AIFM1	28.62	28.43	1.35
AKT1	25.03	25.57	−1.23
APAF1	26.75	27.73	−1.67
BAD	26.45	27.26	−1.48
BAG1	29.99	30.53	−1.23
BAG3	28.67	28.83	1.06
BAK1	28.57	29.22	−1.33
BAX	25.49	25.47	1.2
BCL10	26.7	26.64	1.23
BCL2	27.93	28.5	−1.26
**BCL2A1**	35	31.65	**12.04**
BCL2L1	27.07	27.77	−1.38
**BCL2L10**	35	33.16	**4.23**
BCL2L11	29.39	28.92	1.64
BCL2L2	26.36	27.02	−1.34

BFAR	26.15	26.31	1.06
BID	27.35	27.71	−1.09
BIK	31.33	31.96	−1.31
BIRC2	25.08	25.13	1.14
**BIRC3**	32.62	28.83	**16.34**
BIRC5	30.28	31.45	−1.91
BIRC6	25.79	25.95	1.06
BNIP2	25.94	26.47	−1.22
BNIP3	24.93	25.47	−1.23
BNIP3L	24.57	25.51	−1.62
BRAF	28.18	28.98	−1.47
**CASP1**	30.98	26.03	**36.5**
CASP10	33.21	32.91	1.45
**CASP14**	35	33.58	**3.16**
CASP2	26.32	27.37	−1.75
CASP3	25.66	26.48	−1.49
**CASP4**	33.78	30.89	**8.75**
**CASP5**	35	32.93	**4.96**
CASP6	26.59	27.56	−1.66
CASP7	28.63	27.49	2.6
**CASP8**	31.78	29.47	**5.86**
CASP9	26.72	27.64	−1.6
CD27	33.21	33.36	1.06
**CD40**	35	32.59	**6.28**
CD40LG	32.25	33.14	−1.57
**CD70**	34.53	32.46	**4.96**
CFLAR	27.01	27.15	1.07
CIDEA	35	33.73	2.85
CIDEB	26.93	27.38	−1.16
**CRADD**	28.99	31.15	**−3.78**
CYCS	29.07	29.62	−1.24
DAPK1	27.59	28.71	−1.84
DFFA	26.9	27.75	−1.53
DIABLO	26.34	26.7	−1.09
FADD	27.24	27.27	1.16
FAS	26.84	26.22	1.82
FASLG	35	35	1.18
GADD45A	29.29	27.97	2.95
**HRK**	29.66	27.26	**6.23**
IGF1R	25.66	26.93	−2.04
IL10	34.75	35	−1.01
LTA	32.4	31.26	2.6
LTBR	35	35	1.18
MCL1	24.06	23.93	1.29
NAIP	24.29	25.39	−1.82
NFKB1	29.12	28.14	2.33
NOD1	28.48	28	1.65
NOL3	28.81	29.65	−1.52
PYCARD	30.45	30.25	1.36
RIPK2	28.66	28.65	1.19
**TNF**	35	32.17	**8.4**
TNFRSF10A	33.8	33.3	1.67
TNFRSF10B	26.71	25.92	2.04
TNFRSF11B	28.86	29.21	−1.08

TNFRSF1A	26.1	26.25	1.06
**TNFRSF1B**	35	32.89	**5.1**
TNFRSF21	27.95	27.89	1.23
TNFRSF25	28.63	29.61	−1.67
**TNFRSF9**	35	31.9	**10.13**
**TNFSF10**	34.31	27.85	**103.97**
TNFSF8	35	35	1.18
TP53	25.32	25.17	1.31
TP53BP2	25.29	25.99	−1.38
TP73	35	35	1.18
TRADD	31.55	32.49	−1.62
TRAF2	28.13	28.2	1.13
TRAF3	27.69	27.92	1.01
XIAP	25.84	26.64	−1.47

Table S3. TBEV-induced differential expression of autophagic genes in human neuronal/glial cells. (PCR array data, 72hpi). Up- and down-regulated genes (threshold of 3) are indicated in bold and underscored.

Gene symbol	Ct		Fold Regulation
	NI	TBEV	
AKT1	35.00	35.00	−1.48
AMBRA1	27.75	26.62	1.48
APP	23.53	23.42	−1.37
ATG10	28.97	28.98	−1.49
ATG12	25.70	25.41	−1.21
ATG16L1	27.63	26.99	1.05
ATG16L2	29.24	29.05	−1.30
ATG3	25.60	25.37	−1.26
ATG4A	28.43	28.53	−1.59
ATG4B	26.51	26.13	−1.14
ATG4C	26.74	27.24	−2.09
ATG4D	27.73	27.27	−1.08
ATG5	26.23	26.02	−1.28
ATG7	27.15	27.02	−1.35
ATG9A	27.94	27.37	1.00
ATG9B	34.59	33.01	2.02
BAD	27.05	26.54	−1.04
BAK1	28.85	28.13	1.11
BAX	25.87	24.97	1.26
BCL2	28.03	27.82	−1.28
BCL2L1	27.78	27.43	−1.16
BECN1	27.21	26.79	−1.11
BID	27.47	26.92	−1.01
BNIP3	25.00	24.99	−1.47
CASP3	25.79	25.97	−1.68
**CASP8**	31.46	29.08	**3.52**
CDKN1B	25.11	24.04	1.42
CDKN2A	28.67	27.69	1.33
CLN3	27.60	27.39	−1.28
CTSB	25.67	25.65	−1.46
CTSD	25.46	24.90	−1.00
**CTSS**	31.86	28.58	**6.56**
CXCR4	25.17	25.46	−1.81
DAPK1	28.14	28.19	−1.53

DRAM1	29.74	27.74	2.70
DRAM2	26.72	26.70	−1.46
EIF2AK3	27.75	26.11	2.11
EIF4G1	26.11	25.08	1.38
ESR1	34.41	34.76	−1.89
FADD	27.86	26.76	1.45
FAS	26.83	25.47	1.73
GAA	27.41	27.09	−1.19
GABARAP	23.33	23.27	−1.42
GABARAPL1	26.73	26.36	−1.15
GABARAPL2	23.58	23.79	−1.71
HDAC1	26.23	26.14	−1.39
HDAC6	26.37	26.03	−1.17
HGS	28.95	28.14	1.18
HSP90AA1	23.35	22.57	1.16
HSPA8	22.27	21.82	−1.08
HTT	27.47	26.85	1.04
IFNG	35.00	35.00	−1.48
IGF1	33.50	30.86	4.21
INS	35.00	35.00	−1.48
IRGM	35.00	35.00	−1.48
LAMP1	24.81	24.45	−1.15
MAP1LC3A	26.55	26.50	−1.43
MAP1LC3B	24.48	24.46	−1.46
MAPK14	26.70	26.39	−1.19
MAPK8	24.95	25.54	−2.23
MTOR	26.60	26.21	−1.13
NFKB1	29.38	27.05	3.40
NPC1	27.70	27.11	1.02
PIK3C3	25.97	25.82	−1.33
PIK3CG	35.00	35.00	−1.48
PIK3R4	27.56	27.21	−1.16
PRKAA1	25.96	25.80	−1.33
PTEN	25.26	24.79	−1.07
RAB24	30.11	29.05	1.41
RB1	25.66	25.41	−1.24
RGS19	29.85	29.02	1.20
RPS6KB1	26.14	25.82	−1.19
SNCA	26.65	27.10	−2.02
SQSTM1	24.30	23.02	1.64
TGFB1	27.78	27.41	−1.15
TGM2	33.49	30.85	4.21
TMEM74	32.20	30.06	2.98
TNF	34.53	31.45	5.71
TNFSF10	35.00	27.31	139.49
TP53	25.48	24.49	1.34
ULK1	27.85	27.87	−1.50
ULK2	26.26	26.27	−1.49
UVRAG	29.14	27.63	1.92
WIPI1	27.87	27.81	−1.42
